# Response to the letter to the editor: two cases of subacute thyroiditis after different types of SARS-CoV-2 vaccination

**DOI:** 10.20945/2359-3997000000569

**Published:** 2022-11-28

**Authors:** Hayri Bostan, Ilknur Ozturk Unsal, Muhammed Kizilgul, Umran Gul, Muhammed Erkam Sencar, Bekir Ucan, Erman Cakal

**Affiliations:** 1 University of Health Sciences Diskapi Yildirim Beyazit Training and Research Hospital Department of Endocrinology and Metabolism Ankara Turkey Department of Endocrinology and Metabolism, University of Health Sciences, Diskapi Yildirim Beyazit Training and Research Hospital, Ankara, Turkey

**DEAR EDITORS AND COLLEAGUES,**

We would like to thank Mungmunpuntipantip and Wiwanitkit for their comments and contributions to our article “Two cases of subacute thyroiditis after different types of SARS-CoV-2 vaccination” (1). They commented on the possible mechanisms of vaccine-associated subacute thyroiditis (SAT) and emphasized that subclinical thyroid disorders in the pre-vaccine period should be excluded. We do agree that the exclusion of subclinical thyroid problems is a factor that strengthens the possible relationship. We would like to emphasize that these patients were euthyroid in their routine follow-up visits before the vaccination. In our article, we mentioned that both cases did not have any known thyroid or autoimmune disorder before the coronavirus disease 2019 (COVID-19) vaccination. However, recently, cases of SAT, Graves' disease (GD) relapses, and even conversion from Hashimoto's disease to GD have been reported after the COVID-19 vaccination (2,3). This raises the question that vaccination may be a triggering factor for relapse in patients with pre-existing autoimmune/inflammatory thyroid disorders.

Immunological mechanisms, such as molecular mimicry, epitope spreading, bystander activation, and adjuvant effect, have been postulated in COVID-19 vaccine-associated thyroid disorders (2–4). Mungmunpuntipantip and Wiwanitkit speculated that post-vaccine hyperviscosity may be another mechanism that causes abnormal thyroid hormone levels. However, both the absence of a symptom suggesting hyperviscosity in our cases and the recurrent thyroid diseases reported after vaccination against COVID-19, highlights possible immunological mechanisms. In addition, previously, Tamagna and cols. stated that increased T4 levels in a patient with hyperviscosity syndrome may be due to the interference of increased protein concentrations (5). In conclusion, although hyperviscosity may explain some thromboembolic events associated with COVID-19 and its vaccination, we believe that further evidence is needed to link it with autoimmune/inflammatory thyroid diseases.
